# Ultrafast Optical Properties of Dense Electron Gas in Silicon Nanostructures

**DOI:** 10.1007/s11468-013-9658-z

**Published:** 2013-12-28

**Authors:** A. Sieradzki, M. Basta, P. Scharoch, J.-Y. Bigot

**Affiliations:** 1 Institute of Physics, Wrocław University of Technology, Wybrzeże Wyspiańskiego 27, 50-370 Wroclaw, Poland; 2 Institut de Physique et Chimie des Matériaux de Strasbourg, UMR 7504, CNRS, Université de Strasbourg, BP 43, 23 rue du Loess, 67034 Strasbourg Cedex 02, France; 3 Department of Nanotechnology, Wroclaw Research Centre EiT+, ul Stabłowicka 147, 54-066 Wrocław, Poland

**Keywords:** Pump–probe spectroscopy, Carrier dynamics, Electron–hole plasma, Silicon nanostructure

## Abstract

We investigate the ultrafast dynamics of carriers in a silicon nanostructure by performing spectrally resolved femtosecond spectroscopy measurements with a supercontinuum probe. The nanostructure consists of a 158-nm-thick crystalline Si layer on top of which a SiO_2_ passivation layer leads to a very high quality of the Si surface. In addition, a dielectric function approach, including contributions from a Drude part and interband transitions, combined with the Transition Matrix Approximation is used to model the photogenerated carrier dynamics. The spectrotemporal reflectivity reveals two mechanisms. First, an electron–hole plasma is created by the pump pulse and lasts for a few picoseconds. Importantly, its spectral signature is either a positive or a negative change of reflectivity, depending on the probe wavelength. This is complementary to the already reported results obtained with degenerate frequency measurements. The second mechanism is a thermal diffusion of carriers which occurs during several hundreds of picoseconds. The overall dynamics at short and long delays in the whole visible spectrum is well explained with our model which shows that the main contribution to the reflectivity dynamics is due to the Drude dielectric function. The observation of this predominance of free carriers requires both a long lived high density of carriers as well as a little influence of surface scattering as provided by our thin crystalline Si layer with passivated Si/SiO_2_ interface.

## Introduction

The developing technology of semiconductor devices at the nanoscale and the complexity of the devices themselves still requires to investigate the carrier dynamics on an increasingly shorter timescale and over a broad range of photon energies. In particular, for a device made of an active crystalline layer, the electronic and thermal properties depend not only on the layer itself but also on its surface or interface with a buffer layer. In the case of crystalline silicon, carriers can diffuse over a distance of 10 nm in 100 fs. Therefore, investigating the carrier properties in a Si layer of ∼100 nm requires simultaneously to generate and detect the carrier distributions on an ultrafast time scale and with a controlled surface, still compatible to technological requirements of ambient air pressure and temperature. The ideal situation, as implemented in the present work, is therefore to combine ultrafast pump–probe spectroscopy, using femtosecond laser pulses, with a well-suited sample elaboration approach like the interface passivation in the case of Si.

The pump–probe femtosecond technique has been used over the past two decades [1-6] to investigate the photoexcited carrier dynamics in silicon. As it is well known [7], in a bulk semiconductor excited with a femtosecond optical pulse, as the system evolves toward equilibrium, there is a momentum and energy relaxation [8]. Momentum relaxation occurs on a few tens of femtoseconds scale via elastic and inelastic scattering [9]. On a similar time scale, the carrier–carrier scattering due to the Coulomb interaction leads to the thermalization of the electrons and holes to a Fermi–Dirac distribution [10]. Depending on the excess energy of the carriers on their respective conduction bands, this thermalization process is also assisted by carrier–phonon scattering. Meanwhile, additional processes specific to each considered semiconductor can take place, like the Auger recombination [11] or the intervalley scattering [12]. Importantly, the electrons and holes energy and momentum relaxation combined with their spatial diffusion result in a space and time dependent excited carrier density *N*(*r*,*t*), with a typical time scale of the order of 10 ps. During this time scale, the energy transfer to the lattice is sufficiently important so that its temperature *T*(*r*,*t*) also depends on time and space via the diffusion of heat, within a time scale of a few hundreds of picoseconds. For modeling purposes, the two processes of carrier *N*(*r*,*t*) dynamics and lattice temperature *T*(*r*,*t*) diffusion can be considered, to a good approximation, as independent, regarding their initial values. In other words, it is reasonable to assume that the spatial temperature distribution left by excited carriers after they have relaxed forms an initial condition for the process of heat diffusion.

Interestingly, in the case of silicon, the carrier dynamics has been studied mostly from the point of view of high densities of carriers during the first picosecond. At low densities, the excited carriers form a nearly ideal gas of noninteracting particles. As the density raises up to 10^22^ cm^−3^, the carrier dynamics is well described by the theory of the electron–hole liquid (EHL) [13]. Moreover, at higher carrier densities (2 × 10^23^ cm^−3^), a phase transition from a crystalline solid to a liquid phase may occur [14]. The regime of intermediate densities, where an electron–hole plasma is present, has also been studied [5-7], but so far, the overall dynamical regime from the plasma formation until the heat diffusion takes place has not been studied with details. In the present work, we investigate the carrier dynamics in silicon in a time scale covering both processes of carrier dynamics and heat diffusion, with densities high enough so that the initial excitation regime corresponds to the formation of an electron–hole plasma. Toward that purpose, and complementary to former works [5-7], we have performed time dependent reflectivity Δ*R*/*R*(*λ*,*t*,*E*_abs_) measurements, as a function of wavelength in a broad band visible spectrum, for a long temporal scale (up to 800 ps), and as a function of absorbed pump density of energy *E*_abs_. The samples used are designed so that the electron–hole plasma is confined in a thin Si layer, with little influence of surface scattering effect. The complex observed spectrotemporal dynamics is well explained with a dielectric function that depends on *N*(*r*,*t*) and *T*(*r*,*t*), including both intraband and interband transition processes. Interestingly, our analysis shows that the dynamics is mostly affected by intraband processes corresponding to a Drude-like behavior of the semiconductor.

## Experiment

**Fig. 1:**
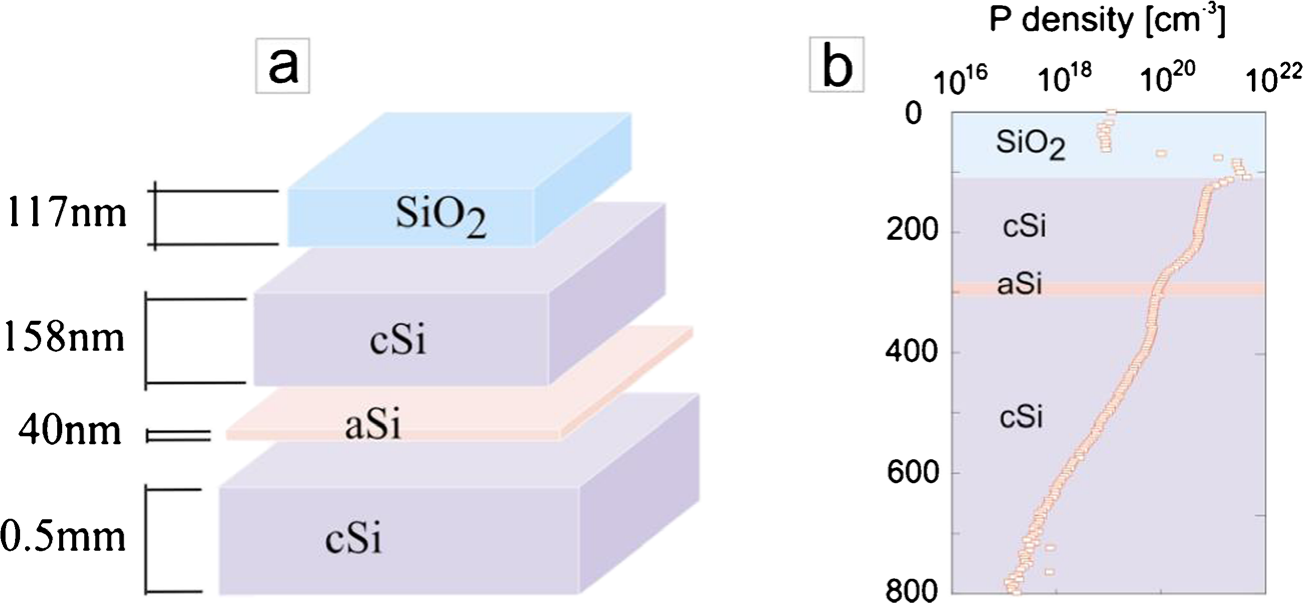
**a** Schematic view of the sample: *cSi* crystalline Si and *aSi* amorphous Si. **b** Shows the P doping distribution in the structure, as a function of the layer and depth measured by secondary ion mass spectroscopy

Our study was conducted on a structure, shown in Fig. 1a, originating from the layered silicon wafer that was P-doped by implantation. The inhomogeneous P doping distribution varies by two orders of magnitude along the distance of 200 nm for implanted–diffused samples. The dopant density profile is shown in Fig. 1b. After the implantation process, the sample was subjected to the thermal oxidation (passivation), with the thickness of the passivation buffer layer equal to ≈200 nm, to guarantee a good quality surface with the desired electronic properties. The passivation buffer layer was partially etched by reactive ion etching (RIE), leaving only a ≈10-nm-thick SiO_2_ layer. Next, another ≈117 nm of SiO_2_ was deposited on top of the 10-nm-thick SiO_2_ buffer layer. There is an amorphous silicon layer aSi in the structure, embedded at a depth of about 158 nm from the interface cSi/SiO_2_ [15], where cSi denotes a crystalline silicon phase.

The time-resolved measurements of the carrier dynamics were carried out using a femtosecond pump–probe technique, in which a short pump pulse excites carriers and a time-delayed probe pulse measures the resulting change in reflectivity as a function of the pump–probe delay time. The optical pulses were generated using an amplified titanium:sapphire laser system operating at a repetition rate of 5 kHz. The pump beam, 150 fs in duration, has a central frequency doubled at 397 nm (about 3.12 eV) using a beta barium borate (BBO) nonlinear crystal. The probe pulses with broad band spectrum were produced by femtosecond white light continuum generation on a thin sapphire plate. The probe beam reflected from the sample was dispersed by a grating in a monochromator and detected by a CCD camera. A band pass filter was placed in front of the detector in order to suppress all the side contributions from the pump and the fundamental beams. Because the supercontinuum pulse is chirped, the recorded reflectivity data have been time-corrected by performing a conformal mapping in the time frequency plane. To determine the chirp in the continuum pulse, we performed two-photon absorption on ZnSe using the same pump and probe pulses. Apart from the supercontinuum beam, we performed also single wavelength probe beam reflectivity measurements.

In both types of experiments, for constant absorbed energy *E*_abs_ of the pump pulse, the reflectivity *R*(*t*, *λ*, *E*_abs_) of p-polarized probe pulses, with an angle of incidence on the sample of 20°, was measured as a function of time delay *t* for chosen wavelengths *λ*. The changes in absorbed energy were achieved by varying the pump intensity. The quantity of interest is the dynamic differential reflectivity (Δ*R*/*R*) = [*R*(*λ*,*E*_abs_)–*R*(*λ*,*E*_abs_ = 0)]/*R*(*λ*,*E*_abs_ = 0), where *R*(*λ*,*E*_abs_ = 0) denotes the reflectivity of the unexcited sample. For measurements of the transient reflectivity, the resulting change in energy of the spectrally filtered probe beam was detected using lock-in amplifiers. For purposes of noise reduction, a separate reference beam was split off the probe beam prior to its interaction with the sample. To correct the pump–probe signal for intensity fluctuations in the continuum at the selected probe wavelength, the signal was normalized by the reference amplitude for each time delay, providing an accuracy of 3 × 10^−4^. In this study, all the measurements were carried out at room temperature. Let us emphasize that the optical pumping remained below the melting threshold [16]. A precise control of the spot size of the pump beam (diameter 100 μm) on the sample during the measurements allowed for an accurate determination of the absorbed fluence. The maximum power of the beam was 1.4 mJ cm^−2^. Two time scales for the dynamical reflectivity are of main interest and named hereafter short (−1 ps → 5 ps) and long (−20 ps → 400 ps) time behaviors.

## Results

**Fig. 2:**
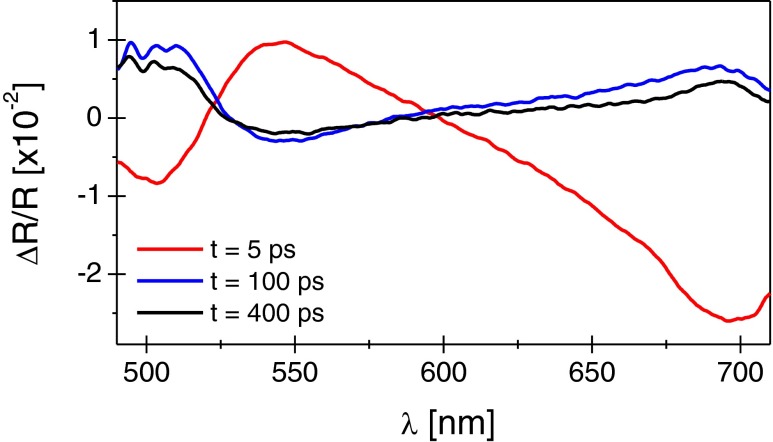
Spectral variation of the differential reflectivity for three temporal delays

**Fig. 3:**
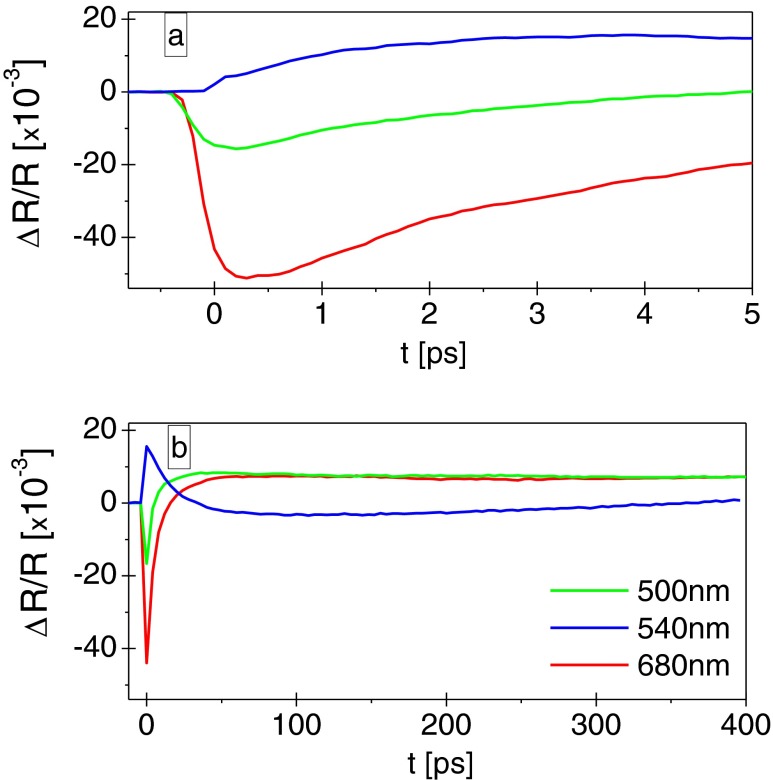
Temporal variation of the differential reflectivity at short **a** and long **b** time delays for three different wavelengths

Figure 2 shows measured spectral differential reflectivity in the probe wavelengths range 490–710 nm for three delay times 5, 100, and 400 ps. The time dependent reflectivity spectrum is complex. For short delays (up to 30 ps), two regions can be distinguished. In the analyzed wavelength range, below 530 nm and above 610 nm, a decrease in the reflectivity is observed, whereas in the range 530–610 nm, the reflectivity change is distinctly positive. The position of the zero crossing points varies when changing the time delay. The spectral range for which the differential reflectivity is positive spreads out reaching a maximum at less than10 ps. The recovery time from the maximum negative change in reflectivity depends on the wavelength as discussed below in Fig. 3. After 80 ps, the spectral shape of the signal is changed so that for the energy range where the differential reflectivity Δ*R*/*R* was negative, it becomes positive and where Δ*R*/*R* was positive, it becomes slightly negative. At longer time delays, the recovery to the static value in the whole spectral range is observed.

Figure 3 shows both a short- and a long-time behavior of reflectivity following the pump pulse excitation for three chosen wavelengths. There are two distinct spectral regions, corresponding to the positive and negative reflectivity changes of Δ*R*/*R*. For *λ* out of the 530–610 nm range, the negative change in reflectivity Δ*R*/*R* recovers with different time constants until it changes sign, also at different times, depending on the wavelength (shorter times for shorter wavelengths). Afterwards, the positive Δ*R*/*R* slowly recovers toward equilibrium over several hundreds of picoseconds. For the wavelength 540 nm, as well as in the spectral range 530–610 nm where Δ*R*/*R* is first positive, the differential reflectivity reaches a maximum in the first few picoseconds. It then decreases crossing the zero at 40 ps. At later time, Δ*R*/*R* slowly decreases and after 120 ps it starts to recover. The time constants τ_a_ as well as the zero crossing point are both wavelength dependent (not shown here).

## Discussion

When discussing the transient reflectivity in the pump–probe experiment, one should be aware of various and complicated underlying physical mechanisms, so we start the discussion from some general notions. First, a high-energy laser pulse, partially reflected/scattered from the surface, excites the carriers with a given distribution in space, on a ∼100 fs time scale. Then, the process of carrier energy scattering and recombination joined with diffusion takes place, over a few picosecond time, with a corresponding time space density distribution. The reflectivity in that time interval is affected mainly by the free intra and interband carrier absorption, including the Γ → *X* intervalley scattering, both limited by the Pauli blocking mechanism. Finally, after the excited carriers have been relaxed, the energy left and distributed in the form of heat begins to diffuse, resulting in a different time-space temperature distribution, with a characteristic time scale of a few hundred of picoseconds. For high-energy laser pulse, the temperature increase is sufficient to induce a significant increase in equilibrium densities, which in turn influences the reflection. Moreover, for certain sample architectures, the interferences of the probe beam may appear which modify the optical response and make the analysis even more complicated.

A common theoretical approach to discuss various factors determining the transient reflection is to expand to linear order the relative change in reflection coefficient with respect to the changes in refraction and extinction coefficients [5, 6, 8]. Then, the short-time (excited densities) and a long-time (temperature) effects are analyzed by considering the excited density and heat diffusion models. In this work, we propose a different approach, which is based on as adequate as possible numerical modeling and numerical experiments. We adopt the Drude–Lorenz model of the dielectric function [17, 18]:

1 $$ {\varepsilon}_S\left(\omega \right)={\varepsilon}_B-\frac{\omega_P^2}{\omega \left(\omega + i\gamma \right)}+{\displaystyle \sum_j\frac{A_j}{\left({\omega}_{gj}^2-{\omega}^2\right)-i{\varGamma}_j\omega }} $$

in which the first term describes the background dielectric constant of Si crystal, the second term is the Drude term describing the metallic-like response from free carriers (optically or temperature induced), while the third term is the Lorentz oscillator linking the probability of absorption of a photon and subsequent transition of electron from valence to conduction band. We have applied an extended form of the Lorentz oscillator that allowed a precise treatment of the doping distribution and the resulting changes in the dielectric function. The meaning of the parameters is: *ω* and ${\omega}_p=\sqrt{N\ {e}^2/{\varepsilon}_0{m}_e^{*}}$ are the incident and plasma frequencies, *N* is the density of carriers, *m*_e_^*^ = *m*_opt_∙*m*_*e*_-- is the effective electronic mass, *m*_opt_ is the optical mass and *m*_*e*_ is the mass of electron, *ε*_B_-- is the background dielectric function of silicon, *γ* is the plasma damping corresponding to the inverse Drude damping time *τ*_D_, *ω*_*gj*_ is interband transition frequencies, Γ_*j*_ is the damping coefficient, and *A*_*j*_-- is the oscillator strength, and the summation in the third term is over the direct interband transitions. To take into account the interferences that appear in optical response of the structure presented in Fig. 1, we have used the one-dimensional Transition Matrix Approximation [18].

**Fig. 4:**
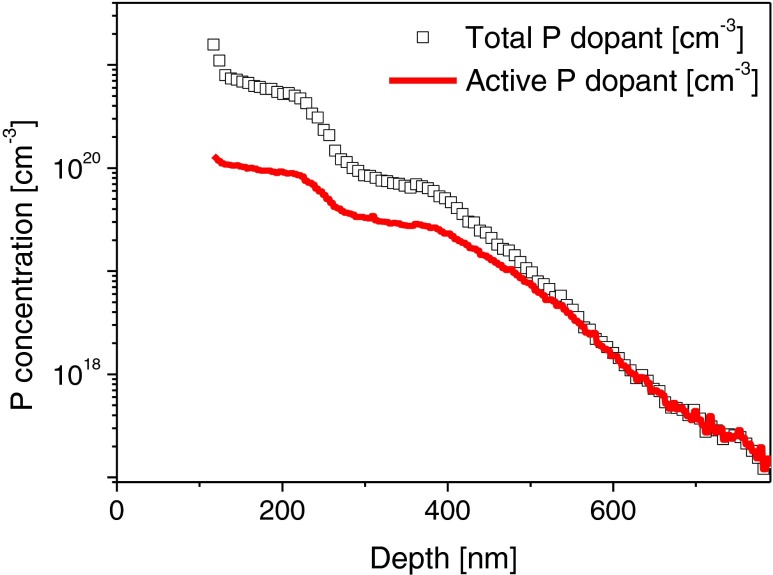
Total vs active dopant concentration in the cSi layer

**Fig. 5:**
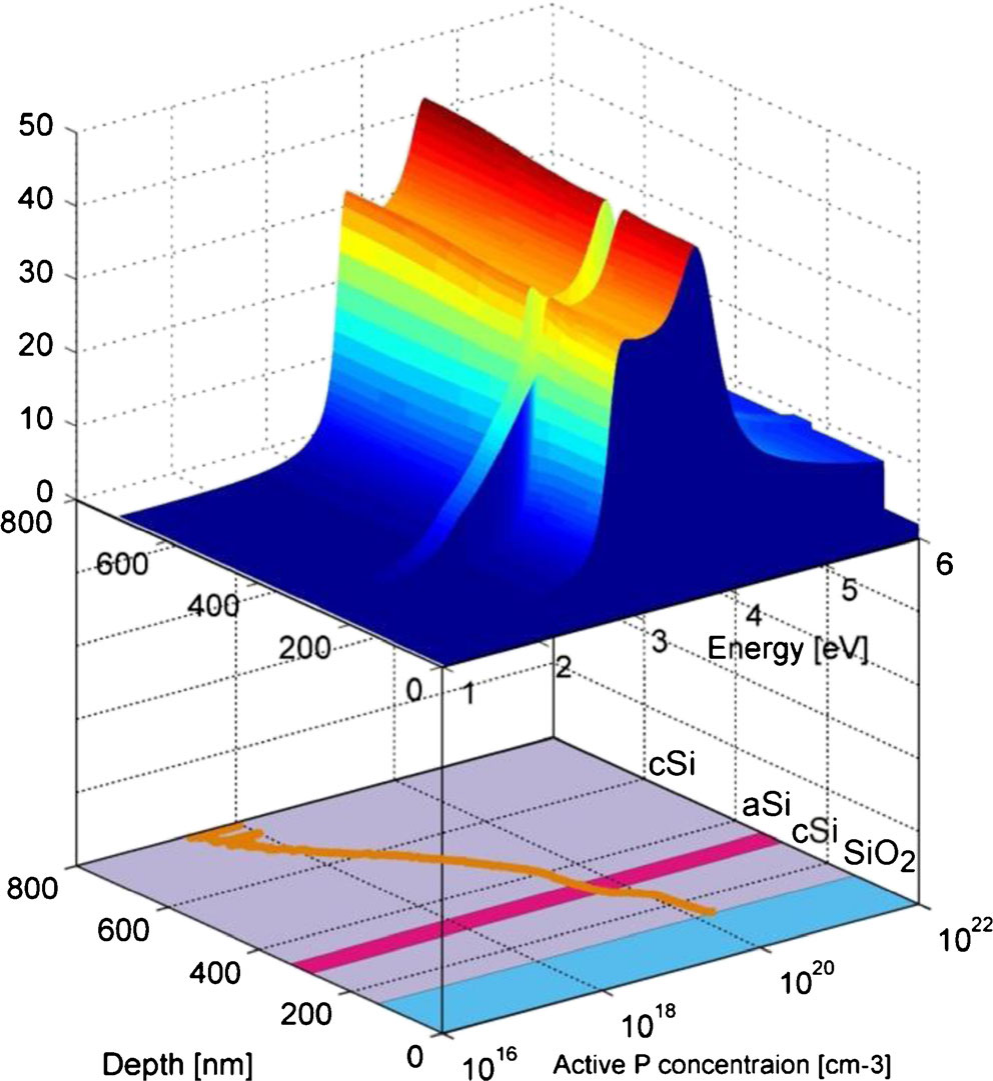
Imaginary part of the dielectric function vs sample depth for accurate structure model (*upper*) and the active P distribution in each layer (*lower*). Thicknesses for each region in this model were SiO_2_ = 117 nm, *upper* cSi = 158 nm, amorphized Si = 40 nm, and *lower* cSi = 400 nm (for precise doping simulation)

**Fig. 6:**
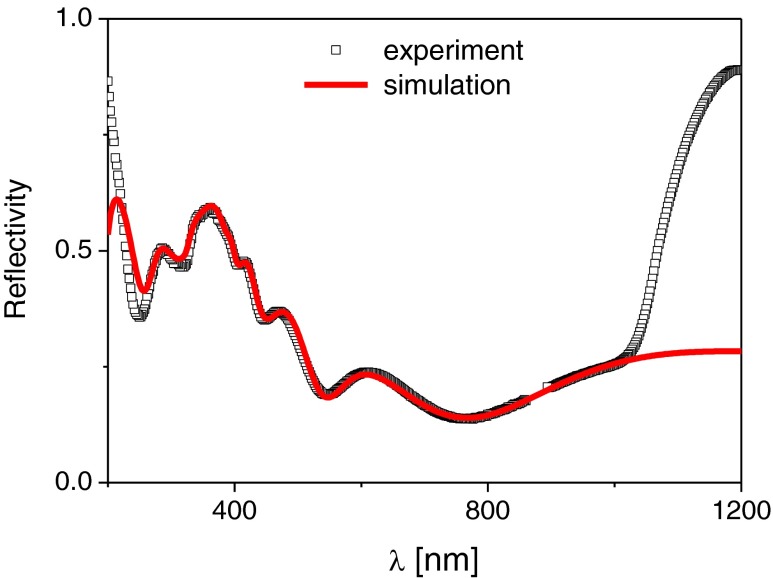
Simulated vs measured static reflectivity

In the first step, we have simulated the static reflection coefficient, matching the experimental and simulation results, with an average deviation less than 1 %. For that purpose, we used the dopant density profile shown in Fig. 1b to evaluate the equilibrium density distribution at room temperature. At 295 K, in room temperature, for P-doped silicon up to concentrations *N*_*d*_ = 10^18^ cm^−3^, the dopants are fully activated. For higher doping concentration, only a fraction of the introduced P atoms are activated, and our evaluation shows that for the highest P concentration, reaching *N*_*d*_ ∼ 10^21^ cm^−3^, only a small fraction of phosphorous is active (about 6 %). This fact is represented by the active dopant concentration (Fig. 4). The equilibrium carriers density allowed us to evaluate the spatially varying dielectric function, taking into account also the amorphized layer and a part of the cSi substrate (Fig. 5). Subsequent calculation of absorption and refraction coefficient has led to the static value of the reflection coefficient which simulated spectral dependence agrees very well with the measurement (Fig. 6). In the simulation, the maximum value of the density in the distribution has been used as the fitting parameter, and for the curve shown in Fig. 6 the corresponding value is *N* = 10^20^ cm^−3^. The values of the other parameters are *m*_opt_ = 0.36, *ω*_*g*1_ = 5.1 × 10^15^ s^−1^, *ω*_*g*2_ = 3.76 × 10^15^ s^−1^, and *ω*_*g*3_ = 4.41 × 10^15^ s^−1^. An interesting observation, both from experiment and from simulation, point of views, is that the interference pattern is present in the whole spectral range, which means that the penetration/observation depth of a probe beam is comparable with the cSi layer thickness (information is gathered from the whole cSi active layer).

In the next step, we focused on simulating Δ*R*/*R*, for the wavelength range 490–720 nm, with a spectral step of 2 nm. Here, as in works [5, 6, 8], two different time scales have been considered. In a short-time scale (up to 20 ps), the simulation has been based on the assumption that the temporal free-carrier distribution plays a dominant role (thus, we discard here the Pauli blocking mechanism, which is not included in the model). The technique is then as follows: only the electronic density is varied while the other parameters are kept constant at their static values. The initial density distribution is assumed to coincide with the absorbed power distribution, calculated from the Poyinting vector and the absorption coefficient. The maximum value of the distribution is the fitting parameter. It is then assumed that during the time evolution, only the maximum value of the density changes and the shape of the profile is preserved. This assumption may seem a coarse one, but we performed many numerical experiments in which other distributions (e.g., Gaussian) have been tested and which showed that the average density rather than the particular shape of distribution affects the reflection. This is true also intuitively since the reflection, which varies with the absorption and refraction, carries an information that depends on the penetration depth of light. Therefore it is an averaging over specific spatial details and shape of the distribution. In our experiment, the penetration depth is comparable with the active cSi layer thickness, because the interference pattern is present. From this point of view, an exact modeling of carrier diffusions seems to be aimless.

**Fig. 7:**
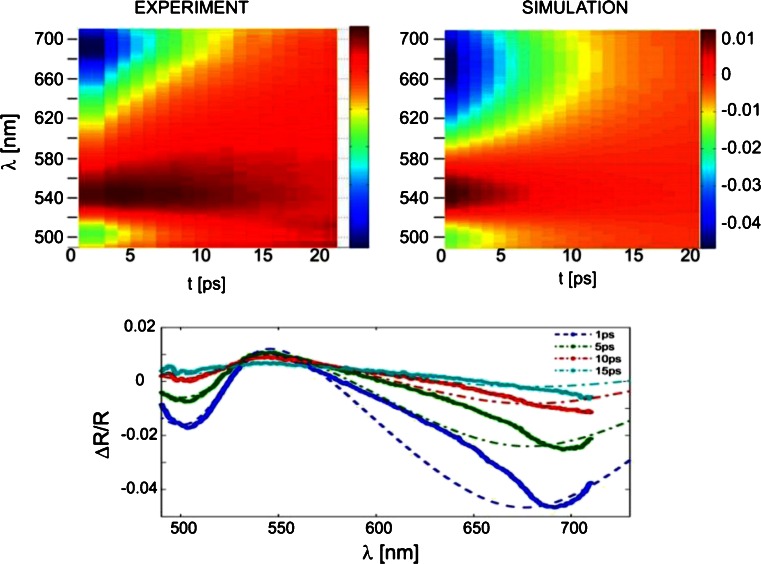
Comparison between experimental (*solid lines*) and simulation (*broken lines*) results of the differential reflectivity spectra for short delay times

**Fig. 8:**
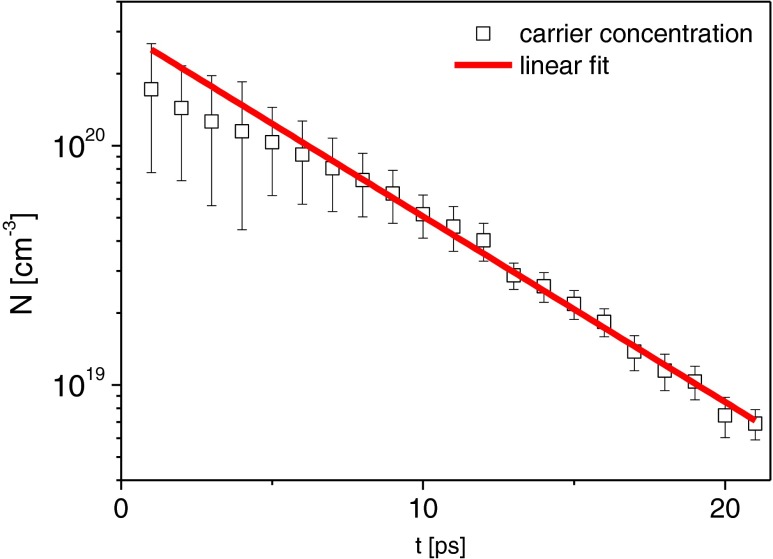
Temporal dependence of the average carrier density used in the model of dielectric function to fit the experimental results. The fitting curve (*red line*) corresponds to an exponential decay

The results of the simulation are shown in Fig. 7. The model reproduces quantitatively the observed spectral dynamics in the spectral range 490–570 nm. An additional information obtained from the simulation is the time-resolved density, which enters the model as a single value parameter and thus can be considered as a kind of penetration depth dependent averaged density. This averaged density exhibits an exponential decay with the time constant *τ* ≈ 10 ps, as shown in Fig. 8.

**Fig. 9:**
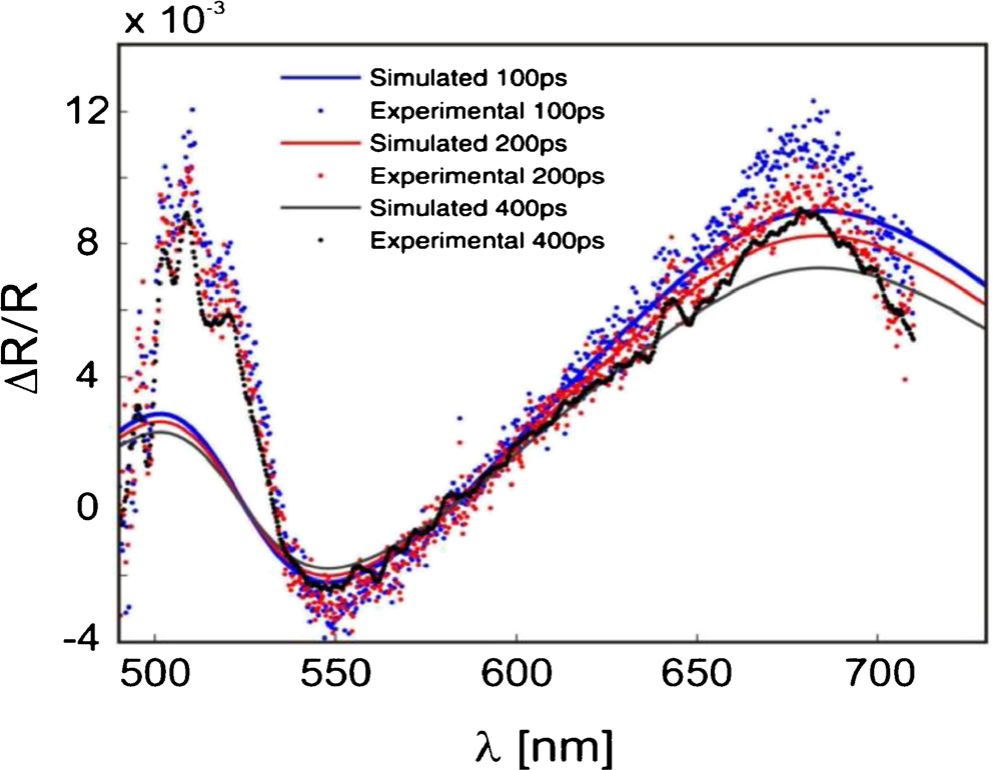
Differential reflectivity spectra at longer time delays; comparison between experimental and simulation results. The fitting parameter is the optical mass *m*_opt_. The electronic density is *N* = 10^20^ cm^−3^

The long-time behavior of reflectivity, as also discussed in [5, 6, 8], is determined by the temperature increase. In our experiment, a simple estimation using the pump pulse energy, reflectivity, predicted absorption volume, and Si-specific heat shows that one can expect an average temperature increase of about 200 K and locally it can be higher. It is high enough to induce significant changes in the equilibrium density. For example, in intrinsic Si for a temperature change from 300 to 500 K, the density increases from 1.5 × 10 to 9.2 × 10^19^ cm^−1^, i.e., almost by 4 orders of magnitude. Here, we deal with the P-doped sample (n-type), with a given dopant density profile (Fig. 1b), and the room temperature value of the excited density is plausible. In our model of optical response, we use the optical mass *m*_opt_ to simulate the long-time behavior of reflectivity. We believe that at higher temperatures, the periodicity of the lattice potential is modified, which is reflected by the increase in the carrier optical mass *m*_opt_. Both parameters are equilibrium density dependent, but the average density itself is kept constant in the simulation, at the value of *N* = 10^20^ cm^−3^. This choice turned out to be sufficient to reproduce successfully the long-time reflectivity behavior. The results are presented in Fig. 9.

The optical mass increases three times more for the shortest time shown (100 ps), which is in agreement with other results published elsewhere [19].

In summary, one can conclude that the high excitation density is essential in the observed change of reflection, both in the short-time scale (high excited density) and in the long-time scale (high temperature increase). The high excitation densities are possible owing to the very good surface quality of our samples, achieved via special surface processing (passivation). The same experiment performed on a very similar sample but with the surface not passivated revealed a standard reflectivity behavior, i.e., the population bleaching mechanism leading to a decrease in the reflectivity.

## Conclusions

The experiments performed in this work give an insight into the carrier dynamics, i.e., the excitation and relaxation processes in specially designed and preprocessed silicon nanostructures. The ultrafast, time, and spectrally resolved reflectivity measurements reveal important facts about the carriers behavior. At short delay times, the standard Pauli blocking mechanism is dominated by the quasimetallic reflection, whereas at longer times, the temperature effects (mainly via the free carriers) play a crucial role. However, the observed phenomena are possible only at high excitation densities; thus, the observations can be made only for a very high quality of illuminated interface, since otherwise the surface scattering and recombination processes make the generation of high densities impossible.
